# Incidence, risk factors, and outcomes of numerical hypotony and choroidal effusion following PRESERFLO MicroShunt implantation

**DOI:** 10.1111/aos.70018

**Published:** 2025-10-21

**Authors:** Emil Nasyrov, Isabel Kommerell, Isabell Schleicher, David A. Merle, Caroline J. Wenzel, Bogomil Voykov

**Affiliations:** ^1^ Centre for Ophthalmology University Hospital Tuebingen Tuebingen Germany

**Keywords:** choroidal detachment, choroidal effusion, hypotony, intraluminal stent, microinvasive bleb surgery, PRESERFLO MicroShunt, primary open‐angle glaucoma, pseudoexfoliative glaucoma

## Abstract

**Purpose:**

To evaluate the incidence, outcomes, and risk factors of numerical hypotony (NH) and choroidal effusion (CE) following standalone PRESERFLO MicroShunt (PMS) implantation.

**Methods:**

Primary open‐angle glaucoma (POAG) and pseudoexfoliative glaucoma (PXG) patients with uni‐ or bilateral surgery at a tertiary university hospital were retrospectively evaluated. Kaplan–Meier estimates of first‐operated eyes were used to analyse the cumulative incidence of NH (intraocular pressure [IOP] of <6 mmHg), CE, and CE requiring intervention. Risk factor analyses were performed using generalised linear mixed models on the patient level, including bilateral cases.

**Results:**

In total, 370 patients (235 POAG and 135 PXG) and 435 eyes (288 and 147) were included. The PXG group had a significantly higher incidence of NH (83.7% vs. 69.4%) and CE (34.6% vs. 20.6%). Interventions for CE were required significantly more in PXG patients (20.7% vs. 7.4%). The median CE duration was significantly longer in the PXG group (14 days vs. 7 days). The following risk factors were identified: PXG (development of NH, CE, and CE requiring intervention); spherical hyperopic refractive error (CE and CE requiring intervention); male sex and higher age (CE); number of preoperative medications (CE requiring intervention), lower preoperative IOP (NH), and higher postoperative IOP drop (CE). The 6‐month visual outcomes were not influenced by the hypotony criteria, but significantly more eyes treated for CE required subsequent bleb revisions.

**Conclusion:**

PXG and hyperopic eyes were at risk for developing postoperative CE requiring intervention. They should be monitored more closely and would benefit from primary intraluminal stenting.

## 1 INTRODUCTION

Hypotony following glaucoma‐filtering surgery is a common, frequently self‐limiting, but potentially sight‐threatening complication (Wang et al., 2019). The World Glaucoma Association's Guidelines on Design and Reporting of Glaucoma Surgical Trials suggest reporting non‐physiological numerical hypotony (NH) defined by a cut‐off IOP of <6 mmHg, along with possible clinical complications of hypotony such as choroidal effusion (CE) and suprachoroidal bleeding that could compromise vision (Shaarawy & Sherwood, 2009).

Microinvasive bleb surgery using the PRESERFLO MicroShunt (PMS) has been developed to minimise the risk of chronic hypotony through the design of an 8.5‐mm‐long stent with a lumen diameter of 70 μm, allowing an outflow resistance of 2–3 (2.6) mmHg at 2 μL/min flow rate based on the Hagen–Poiseuille law, which is consistent with in vitro measurements of 1.301 mmHg/(μL/min) (Ibarz Barberá et al., 2021; Pinchuk et al., 2017). Theoretically and experimentally, the PMS does not provide enough intrinsic outflow resistance to prevent NH, making postoperative pressure control in the early postoperative phase reliant on the resistance from surrounding tissues, such as the sub‐Tenon space and finally by the formation of a filtering bleb (Ibarz Barberá et al., 2021).

A recent Cochrane Database Systematic Review, based on a randomised controlled trial, found a significant risk reduction regarding NH or anterior chamber (AC) shallowing after PMS implantation, compared with standard trabeculectomy (Park et al., 2023). Baker et al. (2021) reported significantly different rates of NH (IOP of <6 mmHg) but did not find significant differences in the rates of clinically significant hypotony, including CE and hypotony maculopathy, or hypotony interventions, between both procedures. A meta‐analysis of retrospective studies did not demonstrate differences in NH or CE rates between both procedures (Khan & Khan, 2024), whereas one registry study reported a higher incidence of both NH and CE following PMS implantation compared with trabeculectomy (Bøhler et al., 2024). These reports highlight that hypotony following PMS implantation is a common complication, with NH reported in up to 39% of cases, CE and AC shallowing in up to 13%, and hypotony maculopathy in up to 7% (Pawiroredjo et al., 2022).

Recently, it has been suggested to reduce hypotony in the early postoperative phase by routinely placing a removable suture (e.g. nylon 10‐0) intraluminally into the PMS (Moktar et al., 2024). Compared with standard PMS implantation, PMS implantation with intraluminal stenting has been reported to reduce hypotony rates in highly myopic eyes as well as a mixed cohort of myopic, primary open‐angle glaucoma (POAG) and pseudoexfoliative glaucoma (PXG) eyes (Lupardi et al., 2023; Verma‐Fuehring et al., 2024).

The long‐term implications of off‐label intraluminal stenting of the PMS remain elusive, with its impact on long‐term outcomes not yet reported. Although impairing outflow might ameliorate the risk of hypotony in the early stages, this could influence bleb maturation, increase fibrosis, and cause higher failure rates over time. Furthermore, in contrast to trabeculectomy and glaucoma drainage device implantation, the rates of clinically significant hypotony presenting with chronic or self‐limiting CE following PMS implantation have not yet been extensively reported. Factors that might indicate the risk for further interventional requirements for hypotony or those that might predict spontaneous resolution of CE have also not been investigated. Therefore, which patients would benefit from primary intraluminal stenting of the PMS and in which patients such a modification might be predictably detrimental remain unclear.

The first part of this study aimed to evaluate the rates and outcomes of NH, CE, and CE requiring intervention, and the second part to analyse the risk factors for these outcomes after standalone unmodified PMS implantation in a large cohort of patients with POAG and PXG.

## 2 METHODS

This retrospective study evaluated consecutive patients with POAG and PXG who underwent standalone unmodified PMS implantation at a single tertiary centre from 2020 to 2023. These included patients with unilateral and bilateral consecutive surgeries. Patients with and without previous glaucoma or cataract surgeries were eligible for inclusion. The first part of the study (time‐to‐event analyses) aimed at describing rates of NH, CE and intervention‐requiring CE, and CE duration during an observation period of up to 6 months. To ensure independence of observations and avoid intra‐individual correlation, potentially biasing standard error estimates and confidence intervals, only the first‐operated eye per patient was included in the time‐to‐event analysis to report robust and comparable rates. We additionally performed sensitivity analysis including bilateral cases. No other exclusion criteria were implemented for the first part of this study. Patients with a follow‐up of fewer than 6 months were censored at the last visit.

In the second part, we exploratorily examined the multivariable association between clinical predictors and binary outcomes of NH, CE, and CE requiring intervention. This was also performed to investigate the association of the main variable of interest (diagnosis of PXG compared with POAG patients) controlling for other variables that could influence outcomes and potentially differed between cohorts. We performed multivariable analyses of both unilateral and bilateral cases, accounting for the correlation between eyes from the same patient. Only eyes with an incomplete follow‐up (<6 months) were excluded if their individual clinical courses did not allow ruling out a possible misclassification of binary outcomes. This involved only 7 (1.6%) out of 442 eligible eyes. The study was conducted in accordance with the tenets of the Declaration of Helsinki. The requirement for patient consent for data to be used in this study was waived by the ethics committee of the University of Tübingen due to its retrospective design (project number 074/2023BO2).

### 2.1 Surgical technique

Surgery was performed by a single experienced surgeon (B.V.) under topical or general anaesthesia, as previously described (Nasyrov et al., 2024).

### 2.2 Perioperative management

Preoperative IOP was assessed under IOP‐lowering medication. Medication was stopped 2 weeks before surgery, and oral acetazolamide 250 mg was started two to three times daily and stopped on the day before surgery. Unpreserved dexamethasone eye drops were administered three times daily 1 week before surgery. Moxifloxacin eye drops were administered four times daily for 2 weeks, starting on the first postoperative day. Unpreserved dexamethasone eye drops were administered five times daily starting from postoperative day 1 and were tapered over 6–8 weeks.

### 2.3 Study outcome measures and baseline characteristics

NH was defined by a cut‐off IOP of <6 mmHg at any visit, regardless of the presence of clinical signs of hypotony (Shaarawy & Sherwood, 2009). The term CE describes the abnormal accumulation of serous fluid in the suprachoroidal space as a sign of clinically significant hypotony. The presence of CE was noted through funduscopic detection of up to four smooth, convex, yellow‐brownish lobes localised in the periphery, delineated by the fixation of the choroid by the vortex veins and possibly involving the posterior pole.

Follow‐ups were conducted on postoperative days 1, 2, 7, and 14 and in postoperative months 3 and 6. Additional visits were scheduled in case of NH or CE or after interventions, at the discretion of the surgeon. These involved a full ophthalmological examination, which included IOP measurement using Goldmann applanation tonometry, assessment of best ‐corrected visual acuitiy (BCVA), slit‐lamp examination, and a complete fundus examination. Patients' characteristics and histories, IOP readings, and other measurements, the presence or absence of CE, and details of interventions for hypotony were retrieved from a proprietary electronic medical record system. Autorefraction was performed using the ARK‐1s autorefractor (NIDEK, Gamagori, Japan) to assess refractive error. The spherical equivalent refractive error of patients with phakia was retrieved from visits where no cataract was noted. Conversely, the refractive error of patients with pseudophakia was obtained from visits where they were phakic.

### 2.4 Postoperative hypotony management

Hypotony was assessed at all postoperative visits. In cases of NH or CE, fluorescein strips were used to exclude leakage. Eyes with peripheral CE were monitored. If CE progressed or involved the posterior pole during the early postoperative visits (days 2 or 7), a viscoelastic with high viscosity (HEALON PRO; Johnson & Johnson Vision) was injected into the AC. At the end of the procedure, the eyes became normotensive. Patients were then monitored overnight and discharged the following day into outpatient care. If CE did not regress or recurred during the first week after the viscoelastic injection, a second injection was performed. If CE was not regressing despite repeated AC injections, intraluminal stenting of the PMS was performed using a 10‐0 nylon suture (Video S1). The intraluminal suture was removed at the discretion of the surgeon only in cases when the IOP was above 16 mmHg in the postoperative follow‐up.

### 2.5 Statistical analysis

Descriptive statistics were used to summarise the demographic and clinical characteristics of patients. For continuous variables, normality was assessed using the Shapiro–Wilk test, which did not indicate a normal distribution of continuous patient characteristics or outcomes. These variables were reported as medians and interquartile ranges (IQRs). Means and standard deviations (SDs) were additionally reported in some cases for illustrative purposes but not used for statistical comparison. Categorical variables were summarised as frequencies and percentages. Comparisons between groups were performed as follows. For continuous variables, the Mann–Whitney *U* test was applied to compare distributions between independent groups when data were non‐normally distributed. For categorical variables, the chi‐squared test or Fisher's exact test was used, as appropriate.

The cumulative incidence of NH and CE, cumulative incidence of intervention for CE, and duration of CE (time from incidence to resolution) were evaluated using Kaplan–Meier survival estimates. To ensure independence of observations and avoid intra‐individual correlation, only the first‐operated eye per patient was included in the time‐to‐event analyses. Sensitivity analysis, including bilateral cases, was additionally performed. Given that the proportional hazards assumption could be violated by the main variable of interest (as CE was significantly more prevalent in the second week in the PXG group) and the assumption was demonstrably violated for at least one variable in the Cox regression model for CE (as assessed by Schoenfeld residuals, *p* < 0.05), we opted not to apply multivariable Cox models for time‐to‐event analyses. Group differences in time‐to‐event were assessed using both the log‐rank test and the Gehan–Breslow–Wilcoxon test, the latter being more sensitive to early differences in survival, especially relevant as non‐proportional hazards were assumed.

For the second part of this study, we performed generalised linear mixed models (GLMMs) with a binomial distribution and logit link function to examine associations between clinical predictors and binary outcomes (NH, CE, CE requiring intervention). To account for intra‐individual correlation (both eyes from the same patient), a random intercept for patient identifier was included. Predictors were selected based on clinical plausibility and prior knowledge, such as those previously reported in the literature, to ensure that relevant confounders are accounted for. These variables were entered simultaneously irrespective of prior univariate analyses. Additionally, exploratory univariate GLMM analyses were performed to highlight candidate variables worth further investigation. Predictors, besides those already selected, with a *p*‐value <0.20 in the univariate analysis were considered for inclusion in multivariable models, provided their inclusion remained theoretically or clinically plausible. However, this approach did only identify one additional potential predictor (oral anticoagulant use), besides those already selected based on clinical rationale and prior knowledge. The following variables were included: age, arterial hypertension, antiplatelet prophylaxis, oral anticoagulation, pseudophakia, refractive error spherical equivalent, diagnosis of PXG (with reference to POAG), sex, age at surgery, preoperative IOP (at time of indication for surgery) or postoperative absolute mean IOP drop within 2 weeks, and the number of preoperative IOP‐lowering medications (Berke et al., 1987; Ercalik et al., 2022; Fannin et al., 2003; Fu et al., 2019; Haga et al., 2013; Iwasaki et al., 2020; Rabiolo et al., 2021; Shin et al., 2020). Medications such as antiplatelet agents or oral anticoagulants were not paused in the perioperative period. Other variables investigated in univariate modelling were prior glaucoma surgical history, postoperative AC haemorrhage, diabetes, preoperative acetazolamide use (prior to 2 weeks to surgery). Crude and adjusted odds ratios (ORs) with 95% confidence intervals (CIs) were calculated by exponentiating the model coefficients and *p*‐values were derived from Wald *z*‐tests. These were reported to indicate the strength and statistical significance of each association. Multicollinearity was assessed using variance inflation factors (VIFs) obtained from corresponding generalised linear models without random effects. A VIF ≥5 was considered indicative of high collinearity.

All analyses were conducted using R version 4.4.0 (R Foundation for Statistical Computing, Vienna, Austria) with the lme4 and survival packages and GraphPad Prism version 10.2.0 (GraphPad Software, Boston, USA). A probability value of *p* < 0.05 was considered statistically significant.

## 3 RESULTS

### 3.1 Study patients

For the first part of this study (time‐to‐event analyses), all 370 eligible patients/first‐operated eyes (135 PXG and 235 POAG) were included in the final analysis. Ten first‐operated eyes in the PXG and 17 in the POAG group had a follow‐up of fewer than 6 months. Table 1 summarises the demographic and clinical characteristics of the groups. The proportions of female patients and of patients with arterial hypertension, and the median age were higher in the PXG group than in the POAG group. The median (interquartile range [IQR]) follow‐up time was 14 [7–25] months and 12 [6–23] months in the POAG and PXG groups, respectively (*p* = 0.3691, Mann–Whitney *U* test).

**TABLE 1:** Patients' demographic and clinical characteristics and surgical history.

Characteristic	Total (*n* = 370)	POAG (*n* = 235)	PXG (*n* = 135)	*p*‐Value
Median age (IQR), year	71 (64–79)	70 (61–78)	74 (66–80)	**<0.0001** ^a^
Mean ± SD age	70.4 ± 10	68.6 ± 10.4	72.3 ± 8.4	
Male sex, %	53.0	54.8	45.2	**0.0237** ^b^
White ethnicity, %	100	100	100	
Right eye, %	48.7	47.4	49.4	0.7455^b^
Pseudophakia, %	64.6	61.3	70.4	0.0904^b^
Arterial hypertension, %	57.6	53.2	65.2	**0.0289** ^b^
Diabetes, %	15.7	15.3	16.3	0.8821^b^
Antiplatelet prophylaxis, %	17.8	17.0	19.3	0.6724^b^
Oral anticoagulant, %	10.5	8.5	14.1	0.1133^b^
Median refractive error (IQR), dpt spherical equivalent	−0.375 (−2.75 to +0.25)	−0.25 (−2.75 to +0.5)	−0.5 (−2.5 to 0)	0.7506^a^
Median preoperative medicated IOP (IQR), mmHg	28 (23–32)	27 (24–32)	28 (23–32)	0.2198^a^
Median number of preoperative medications (IQR)	3 (2–4)	3 (3–4)	3(2–4)	0.1941^a^
Median BCVA (IQR), logMAR	0.2 (0.–0.4)	0.2 (0.1–0.4)	0.2 (0–0.3)	0.4908^a^
**Previous glaucoma procedures, %**				0.2862^c^
None	58.9	60	58.3	
Laser trabeculoplasty	9.7	7.4	11.1	
Angle‐based procedures	6.2	5.9	6.4	
XEN‐45 implantation	11.9	8.9	13.6	
Trabeculectomy	9.2	12.6	7.2	
Glaucoma drainage device implantation	0	0	0	
Cyclo‐/cryoablation	4.1	5.2	3.4	

*Note*: IOP and medications at the time of indication for surgery. The number of medications used was calculated based on the individual active agents.

Abbreviations: IQR, interquartile range; POAG, primary open‐angle glaucoma; PXG, pseudoexfoliative glaucoma; SD, standard deviation.

Bold values *p* < 0.05 were highlighted.

^a^ Statistical tests: Mann–Whitney *U* test.

^b^ Fisher's exact test

^c^ chi‐square test.

**FIGURE 1:**
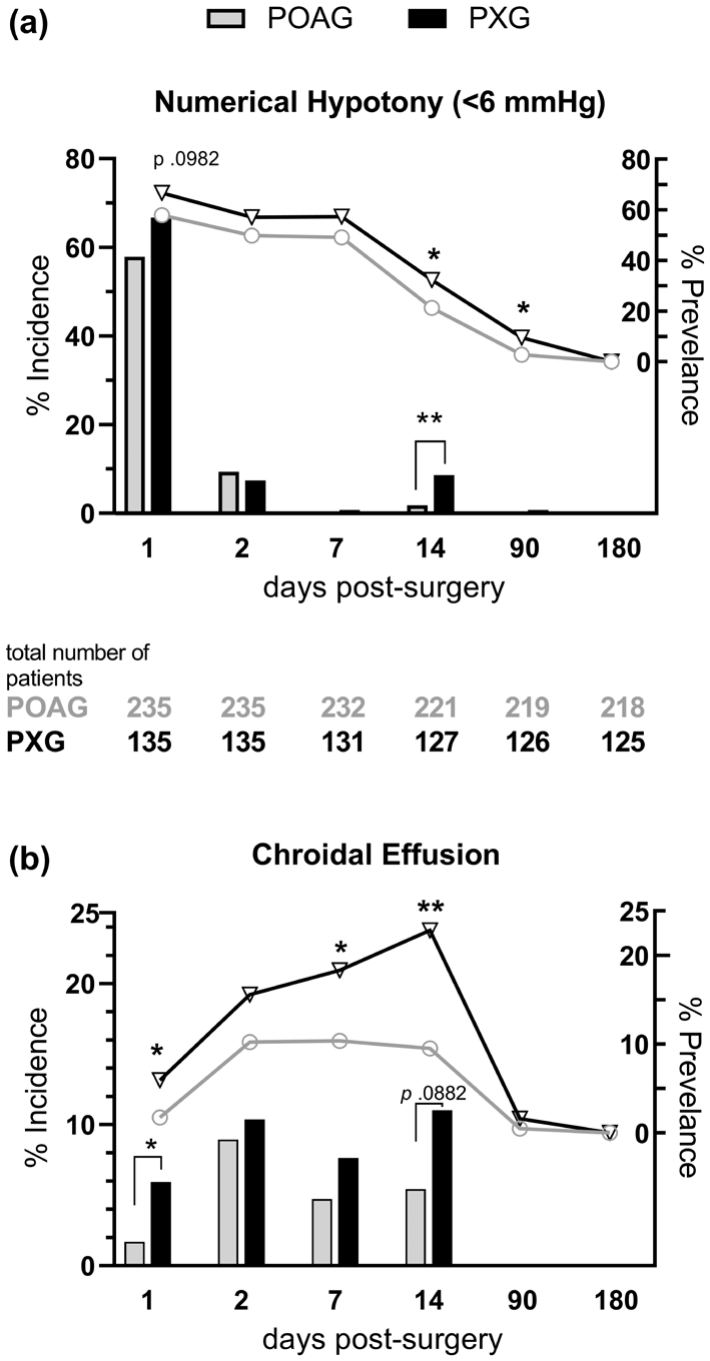
Incidence and prevalence of numerical hypotony and choroidal effusion. The incidence (left *y*‐axis, bars) and prevalence (right *y*‐axis, lines) of numerical hypotony (a) and choroidal effusion (b) are plotted across postoperative visits. The pseudoexfoliative glaucoma (PXG) group is represented by black bars and triangles and the primary open‐angle glaucoma (POAG) group by grey bars and circles. *p < 0.05; **p < 0.01 indicated by Fisher's exact test.

Regarding the inclusion of unilateral and bilateral cases into the second part of this study (multivariable risk factor analyses of binary outcomes), a cut‐off of at least 14 days of postoperative follow‐up, in which either no CE or CE with resolution had to be noticed, was chosen to prevent possible misclassification of binary outcomes. The cut‐off was based on the observation that CE was not found to be incident after 14 days in our study (Figure 1). Incident NH was only seen in two patients after 14 days, and this was deemed negligible. In the final multivariable analyses, 132 PXG and 232 POAG patients/first‐operated eyes (135 PXG and 235 eligible) were included. In bilateral cases, 15 second eyes in the PXG and 56 second eyes in the POAG group (15 and 57 eligible eyes) were additionally included. In the PXG and POAG groups, 10 and 17 of the first eyes, and 1 and 10 of the second eyes, respectively, had a follow‐up of fewer than 6 months.

### 3.2 Incidence and prevalence of NH and CE

Figure 1 shows the incidence and prevalence of NH and CE. The incidence of postoperative NH was highest on day 1 in both groups and indicated by a trend to be higher in PXG compared with POAG patients (66.7% vs. 57.9%; *p* = 0.0982, Fisher's exact test; Figure 1a). On day 14, the incidence of NH was significantly higher in the PXG patients than in the POAG patients (8.7% vs. 1.8%; *p* = 0.0044; Figure 1a). On day 90, NH was noted only in 0.5% and 0.8% of POAG and PXG patients, respectively.

The prevalence of NH was highest on days 1–7 and similar between both POAG and PXG groups (Figure 1a). It steadily declined in both groups from day 7 to day 90 and reached 0% on day 180. However, it was significantly higher in the PXG patients on day 14 (32.3% vs. 21.3%; *p* = 0.0292) and day 90 (9.5% vs. 2.7%; *p* = 0.0102; Figure 1a).

In both groups, incidence of CE was noted at each visit until day 14 in a proportion of patients of both groups, but not on day 90 onward (Figure 1b). The incidence of CE was significantly higher on day 1 in the PXG patients (5.9% vs. 1.7%; *p* = 0.0346) with a trend on day 14 (11% vs. 5.4%; *p* = 0.0882). The incidence of CE in the POAG group was highest on day 2 (8.9% vs. 10.4%; *p* = 0.3489).

The prevalence of CE was highest in the POAG patients on days 2, 7, and 14 (10.2%, 10.3%, and 9.5%), after which it declined to 0.5% by day 90. In contrast, the prevalence of CE increased in the PXG patients from significantly higher rates starting on day 1 to 15.6% on day 2 (*p* = 0.134), to 18.3% on day 7 (*p* = 0.0363), and to its maximum of 22.8% on day 14 (*p* = 0.0013; Figure 1b). On day 90, the prevalence of CE was 1.6% in the PXG patients, comparable to 0.5% in the POAG patients (*p* = 0.3022). CE was not prevalent on day 180 in either group.

### 3.3 Cumulative incidence of NH, CE and CE requiring intervention

**FIGURE 2:**
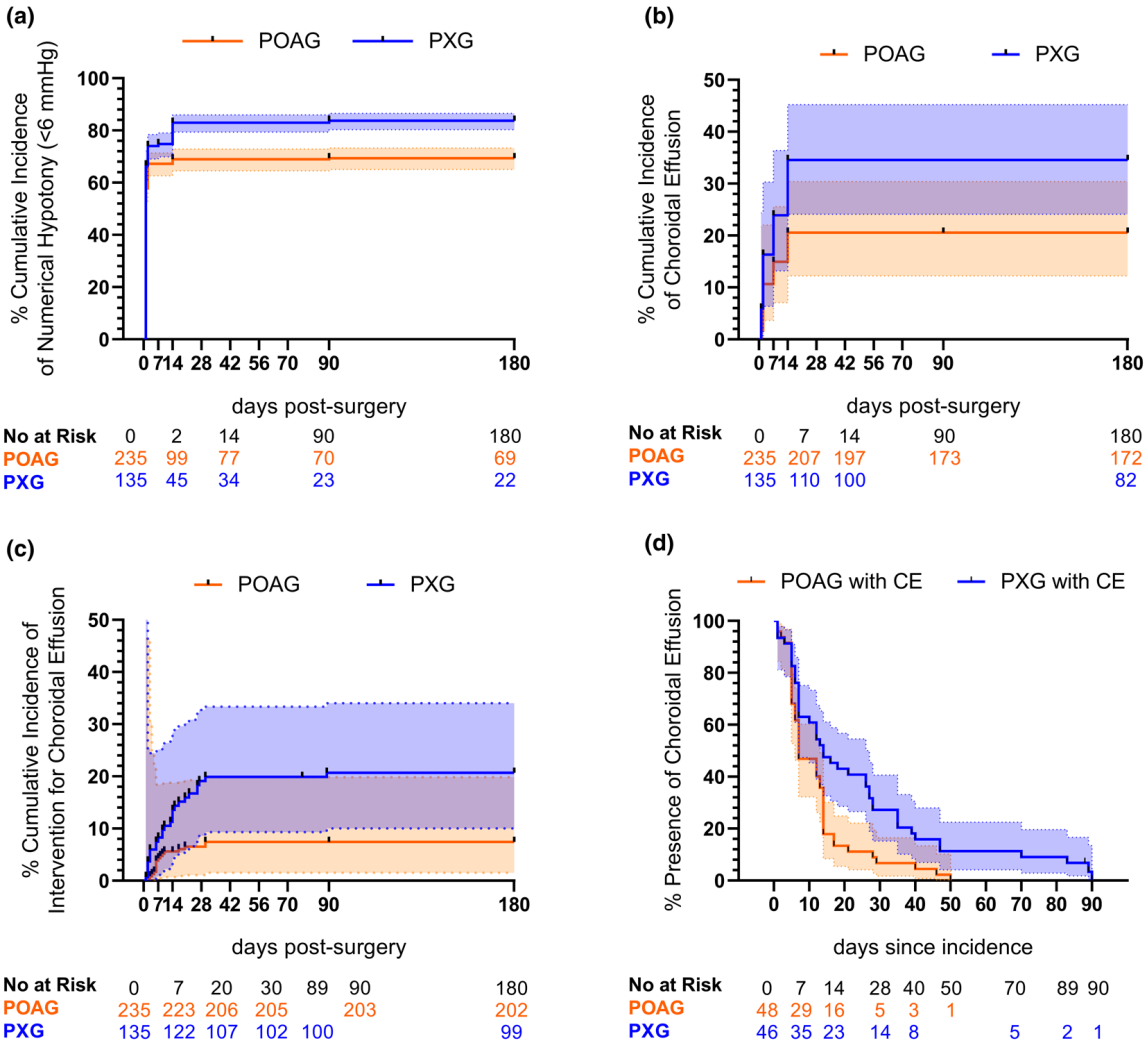
Kaplan–Meier analyses of first‐operated eyes. Kaplan–Meier estimates were used to analyse the cumulative incidence of numerical hypotony (IOP of <6 mmHg) (a), choroidal effusion (b) and choroidal effusion requiring intervention (c) and the duration of choroidal effusion (d) between the first‐operated eyes of patients with primary open‐angle glaucoma (POAG; orange) and pseudoexfoliative glaucoma (PXG; blue). The 95% confidence intervals are represented in lighter orange and blue colours, respectively.

The cumulative incidence of NH was significantly higher in the PXG patients than in the POAG patients (*p* = 0.0051, log‐rank test; *p* = 0.0348, Gehan–Breslow–Wilcoxon test; Figure 2a). It was 83% (95% CI = 79.4–86) and 68.9% (95% CI = 64.5–72.9) after 14 days, respectively. This was 83.7% (95% CI = 80.3–86.6) and 69.4% (95% CI = 65–73.3) after 90 days, respectively. The cumulative incidence of CE was also significantly higher in the PXG group (*p* = 0.003, log‐rank test; *p* = 0.0036, Gehan–Breslow–Wilcoxon test; Figure 2b). It was 34.6% (95% CI = 24.1–45.2) and 20.6% (95% CI = 12.2–30.4) after 14 days in the PXG and POAG groups, respectively. Conversely, the cumulative incidence of CE requiring intervention was 19.9% (95% CI = 9.3–33.4) and 7.4% (95% CI = 1.5–19.9) after 30 days in the PXG and POAG groups, respectively (*p* = 0.0002, log‐rank test; *p* = 0.0003, Gehan–Breslow–Wilcoxon test; Figure 2c). This was 20.7% (95% CI = 10–34) after 90 days in the PXG group (Figure 2c).

Of the eyes that developed CE, 59% (27/46) in the PXG group and 38% (18/48) in the POAG group required at least one viscoelastic injection into the AC (*p* = 0.0625, Fisher's exact test). The median (IQR) number of AC injections was comparable between the PXG and POAG patients with CE that required intervention (2 [1–3] vs. 1.5 [1–2]; range = 1–7 vs. 1–4; *p* = 0.6708, Mann–Whitney *U* test; mean ± SD = 1.96 ± 1.32 vs. 1.72 ± 0.9). Following AC injections, 10.9% (5/46) and 6.3% (3/48) of the PXG and POAG patients required additional intraluminal PMS stenting (*p* = 0.4812, Fisher's exact test) after a median of 59 and 43 days, respectively (*p* = 0.4, Mann–Whitney *U* test).

Both CE and CE requiring intervention were significantly more common in the PXG group than in the POAG group. Therefore, the median (IQR) number of AC injections was significantly higher in the PXG group than in the POAG group in the subanalysis of all eyes with CE (1 [0–2] vs. 0 [0–1]; *p* = 0.0214, Mann–Whitney *U* test; mean ± SD = 1.15 ± 1.34 vs. 0.6 ± 0.98).

### 3.4 Duration and onset of CE

The median duration of CE (onset until resolution) was significantly longer in the PXG eyes than in the POAG eyes (14 days vs. 7 days; ratio = 2; 95% CI = 1.3–3; *p* = 0.0025, log‐rank test; *p* = 0.0215, Gehan–Breslow–Wilcoxon test; Figure 2d). Among the complete cohort and subgroup analyses, there was no significant difference in median duration in patients with CE requiring intervention compared with the patients with spontaneous CE resolution. The comparison between the PXG and POAG patients that required CE intervention indicated a trend towards a longer median duration in the PXG group in the log‐rank test (14 days vs. 13 days; *p* = 0.0616, log‐rank test; *p* = 0.1517, Gehan–Breslow–Wilcoxon test). No difference was found for median duration of CE with spontaneous resolution between the groups (7 days vs. 7 days; *p* = 0.1891; *p* = 0.1301, Gehan–Breslow–Wilcoxon test).

A trend indicated that CE that required intervention occurred earlier than CE with spontaneous resolution in the complete cohort (median = day 2 vs. day 7; *p* = 0.0729, log‐rank test; *p* = 0.0586, Gehan–Breslow–Wilcoxon test) but not in the PXG or POAG subgroups. Between the PXG and POAG patients, no significant differences were found in the onset of CE with spontaneous resolution or CE requiring intervention.

### 3.5 Sensitivity analysis

Sensitivity analysis of time‐to‐event analyses for the cumulative incidence of NH, CE, CE with intervention, and median duration of CE was performed, including bilateral eyes of 72 patients (442 eyes of 370 patients). It found significant differences and similar survival rates between PXG and POAG eyes, as the first‐operated eye per patient analyses depicted in Figure 2 (Figure S1).

### 3.6 Complications

In the POAG group, one eye developed kissing choroids after 2 weeks, which resolved following AC formation and intraluminal PMS stenting. One eye required puncturing of CE on postoperative day 5 and administration of oral prednisolone for 2 weeks. A loss of BCVA from 0.8 logMAR before surgery to 1.2 logMAR after 6 months was noted.

In the PXG group, one eye developed kissing choroids after 2 weeks, which resolved following AC formation. Massive suprachoroidal bleeding was noted in one eye on day 7 despite previous AC formation and required further cut‐down drainage with vitrectomy and silicone oil implantation after 19 days. A loss of BCVA from 0.6 logMAR before surgery to 2.6 logMAR after 6 months was noted. No funduscopic signs of hypotony maculopathy were observed in any patient at any visit. The incidence of suprachoroidal bleeding did not differ between the groups.

### 3.7 Outcomes of BCVA and bleb revision surgery (needlings and incisional)

Among the total cohort that completed the 6 months follow‐up, 14% (48/343) lost two lines or more of BCVA (logMAR) after 6 months. Reasons included the progression of a pre‐existing maculopathy such as epiretinal membrane or age‐related macular degeneration, cataract progression, and worsening of a pre‐existing corneal pathology. Regarding the proportion of patients that lost two or more lines of BCVA after 6 months, no significant differences were found between the eyes with and without NH (13.9% vs. 14.3%; *p* > 0.9999, Fisher's exact test) or between those with and without CE (17.9% vs. 12.6%; *p* = 0.2168). Loss of two lines or more of BCVA was not more common in the eyes with CE that required intervention than in the eyes with spontaneous resolution of CE (19.1% vs. 13.3%; *p* = 0.3412).

The proportion of eyes requiring bleb revisions within the first 6 months and the median number of revisions were also analysed. The bleb revision rate constituted mostly needlings and, to a lesser extent, incisional revisions. There was a non‐significant trend towards a lower bleb revision rate in the eyes with NH than in those without NH (13.1% vs. 20.9%; *p* = 0.0884, Fisher's exact test).

The patients with CE that did not require hypotony intervention had a comparable bleb revision rate to the eyes without CE (14.9% vs. 11.8%; *p* = 0.3496, Fisher's exact test). However, the eyes with CE that required intervention showed a higher rate of bleb revision surgery within 6 months than the eyes with spontaneous resolution of CE (35.7% vs. 14.9%; *p* = 0.0283) and all eyes in the cohort without hypotony intervention (12.3%; *p* < 0.0001).

Among the patients that underwent bleb revision surgery within 6 months (52/343; 15.6%), no difference existed in the median number of revisions between those with and without previous NH or between those with and without previous CE (both 1 vs. 1; *p* = 0.936 and 0.9699, respectively, Mann–Whitney *U* test). However, there was a trend for more bleb revision surgeries in those that had previous CE treated with hypotony intervention than those that had self‐limiting CE (median [IQR] = 2 [1–2] vs. 1 [1–1]; *p* = 0.0657, Mann–Whitney *U* test; mean ± SD = 1.93 ± 1.1 vs. 1.14 ± 0.38).

### 3.8 Risk factor analyses

For the risk factor analyses, bilateral cases, statistically accounting for intra‐individual correlation, were eligible for inclusion. To assess potential selection bias regarding whether the fellow eye was operated on depending on the complications observed in the first eye, contingency table analyses were performed. Analyses did not demonstrate significant differences in the proportion of CE in the first‐operated eye of patients with bilateral surgery compared with those with unilateral surgery in the complete cohort (22.2% vs. 26.2%, *p* = 0.5484; Fisher's exact test) or the PXG and POAG subgroups (26.7% vs. 35%, *p* = 0.7734, and 21.1% vs. 20.2%, *p* = 0.8529, respectively). No significant differences were found regarding the proportion of eyes with CE requiring intervention in the first‐operated eye of patients with bilateral compared with unilateral PMS implantation in the complete cohort (9.7% vs. 12.4%, *p* = 0.6853) or the PXG and POAG subgroups (13.3% vs. 20.8%, *p* = 0.7348, and 8.8% vs. 6.7%, *p* = 0.5677, respectively).

The PXG and POAG groups significantly differed in their baseline characteristics such as age, arterial hypertension, and sex (Table 1). Risk factor analyses using multivariable GLLMs were performed to assess the influence of PXG and other variables on the outcomes of NH, CE, and CE requiring intervention to assess the respective association of each variable with the outcomes (Tables 2, 3, 4). Variable selection was done based on clinical rationale and prior knowledge. Exploratory univariate GLLMs found oral anticoagulant use to be another potential predictor (Table S1), and this was included in the final multivariable GLLMs. The mean postoperative IOP drop (calculated as the mean IOP from postoperative day 1 to 14 subtracted from the preoperative IOP) was assessed for the eyes with CE and CE requiring intervention, but not for those with NH, since the IOP drop was associated with the outcome of NH (IOP of <6 mmHg) and unfit as a predictive variable. Preoperative IOP and postoperative mean IOP drop showed high multicollinearity (VIFs >5); thus, only the latter was chosen for inclusion in the CE and intervention‐requiring CE multivariable GLLMs, and the former for the NH multivariable GLLM. Univariate GLLMs are shown in Table S1. Tables 2, 3, 4 show the adjusted ORs with *p*‐values and 95% CIs for each variable in the multivariable GLLMs.

**TABLE 2:** GLLM for numerical hypotony.

Variable	*p*‐Value	OR	CI low	CI high
Age. years	0.3017200	0.985	0.958	1.013
Male sex	0.9070500	1.027	0.652	1.619
Arterial hypertension	0.8996600	0.969	0.590	1.589
Antiplatelet prophylaxis	0.4817300	1.259	0.663	2.388
Oral anticoagulants	0.0506370	2.605	0.997	6.804
Diagnosis of PXG (reference: POAG)	*0.0074107*	2.075	1.216	3.540
Refractive error, dpt spherical equivalent	0.9977000	1.000	0.948	1.055
Pseudophakia	0.9819700	1.006	0.592	1.711
Number of preoperative medications	0.4715100	1.069	0.891	1.284
Preoperative medicated IOP, mmHg	*0.0233860*	0.964	0.933	0.995

Abbreviations: CI, 95% confidence interval; dpt, dioptre; POAG, primary open‐angle glaucoma; PXG, pseudoexfoliative glaucoma.

The multivariable model for the outcome of NH found that the diagnosis of PXG with reference to POAG (OR = 2.075, 95% CI = 1.216–3.354) and lower preoperative IOP (OR = 0.964 per 1 mmHg, 95% CI = 0.933–0.995) were significant predictors of NH development (Table 2).

The model for CE demonstrated that increasing age (OR = 1.064 per year, 95% CI = 1.019–1.112), male sex (OR = 2.237, 95% CI = 1.209–4.14), the diagnosis of PXG (OR = 2.063, 95% CI = 1.106–3.845), hyperopic refractive error (OR = 1.155 for each 1‐dpt increase in spherical equivalent, 95% CI = 1.042–1.28), and postoperative mean IOP drop (OR = 1.066 per 1 mmHg, 95% CI = 1.019–1.114) were risk factors for the development of postoperative CE (Table 3).

**TABLE 3:** GLLM for choroidal effusion.

Variable	*p*‐Value	OR	CI low	CI high
Age, years	*0.0053163*	1.064	1.019	1.112
Male sex	*0.0103550*	2.237	1.209	4.140
Arterial hypertension	0.3309000	0.730	0.387	1.377
Antiplatelet prophylaxis	0.5245000	1.275	0.603	2.698
Oral anticoagulants	0.7740300	1.144	0.457	2.859
Diagnosis of PXG (reference: PAOG)	*0.0227140*	2.063	1.106	3.845
Refractive error, dpt spherical equivalent	*0.0061930*	1.155	1.042	1.280
Pseudophakia	0.9912300	1.004	0.506	1.993
Number of preoperative medications	0.1283700	1.224	0.943	1.589
Mean postoperative IOP drop (preoperative IOP – mean IOP [days 1, 2, 7 and 14]), mmHg	*0.0051435*	1.066	1.019	1.114

Abbreviations: CI, 95% confidence interval; dpt, dioptre; POAG primary open‐angle glaucoma; PXG, pseudoexfoliative glaucoma.

The model for CE requiring intervention identified the diagnosis of PXG (OR = 3.256, 95% CI = 1.507–7.038), hyperopic refractive error (OR = 1.323 for each 1‐dpt increase in spherical equivalent, 95% CI = 1.134–1.544), and the number of preoperative IOP‐lowering agents to be predictive risk factors (OR = 1.526 per agent, 95% CI = 1.02–2.282; Table 4). The interaction terms between the key variables, such as PXG and age, pseudophakia, refractive error, and sex did not indicate significant interaction effects on any outcome.

**TABLE 4:** GLLM for choroidal effusion requiring intervention.

Variable	*p*‐Value	OR	CI low	CI high
Age, years	0.1037700	1.046	0.991	1.105
Male sex	0.6427500	0.827	0.371	1.844
Arterial hypertension	0.7199600	0.855	0.362	2.017
Antiplatelet prophylaxis	0.1337800	2.065	0.800	5.326
Oral anticoagulants	0.1006900	2.455	0.840	7.177
Diagnosis of PXG (reference: PAOG)	*0.0026787*	3.256	1.507	7.038
Refractive error, dpt spherical equivalent	*0.00037256*	1.323	1.134	1.544
Pseudophakia	0.6876300	1.217	0.467	3.176
Number of preoperative medications	*0.0396860*	1.526	1.020	2.282
Mean postoperative IOP drop (preoperative IOP – mean IOP [days 1, 2, 7 and 14]), mmHg	0.3272000	1.026	0.975	1.079

Abbreviations: CI, 95% confidence interval; dpt, dioptre; POAG primary open‐angle glaucoma; PXG, pseudoexfoliative glaucoma.

## 4 DISCUSSION

Our study found that the PXG patients were more likely to develop both NH and CE following standalone PMS implantation than the POAG patients. NH (IOP of <6 mmHg at any visit) was found in 83.7% and 69.4% of the PXG and POAG patients, respectively. Conversely, clinically significant hypotony with CE was noted in 34.6% of the PXG patients and 20.6% of the POAG patients. PXG and POAG patients that developed CE required intervention with at least one viscoelastic injection in 59% and 38%, and intraluminal PMS suture stenting in 10.9% and 6.3%, respectively. The overall incidence of CE requiring intervention was 20.7% in the complete PXG group and 7.4% in the POAG group. All cases of CE resolved within 3 months after onset. Neither NH nor CE had any influence on the short‐term visual outcomes. However, the eyes with CE that required intervention needed bleb revision surgery (needlings or incisional revision) more often.

A comparison of our rates to previous reports is difficult, especially for NH, due to the differing definitions employed. This might explain why our NH rates are comparable to those reported by Bøhler et al. (2024) (same definition) but higher when a stricter definition is applied (IOP of <6 mmHg on two visits) (Pawiroredjo et al., 2022). Conversely, the rate of CE following PMS implantation was higher in our study than in previous reports (Baker et al., 2021; Bøhler et al., 2024). This might be because, contrary to the quantitative definition used for NH, CE is defined qualitatively. Therefore, including both peripheral and central CE would increase the rate of overall CE compared with that in studies that do not include peripheral CE because of fundus assessment without pupil dilation, for example. This effect was reported in a study assessing CE after Ahmed glaucoma valve (AGV) implantation using wide‐field fundus or 45° central fundus photography (Shin et al., 2020). Additionally, the higher rates of NH, CE, and CE requiring intervention in our study might be explained by the higher number of included patients with PXG, which we found to be an independent risk factor of NH, CE, and CE requiring intervention.

Multivariable GLLM analysis found that lower preoperative IOP was a predictor of postoperative NH. Conceptually, outflow is governed by the pressure gradient between the AC and the subconjunctival space given that flow resistance through the PMS is fixed and aqueous humour viscosity is constant. Eyes with low preoperative IOP may reflect lower steady‐state aqueous humour production or may require only a small gradient to maintain flow, which may predispose them to postoperative NH. Alternatively, ocular structural characteristics such as reduced corneal pachymetry, which has been associated with lower measured IOP, might predispose certain eyes to both lower preoperative and postoperative pressures (Martinez‐de‐la‐Casa et al., 2006).

Besides lower preoperative IOP levels, PXG was the only other risk factor for the development of NH in our study. Pseudoexfoliation (PEX) syndrome is a generalised disorder characterised by a range of extraocular (e.g., vascular) and intraocular manifestation sites such as the trabecular meshwork and ciliary body (Naumann et al., 1998). In vivo studies of aqueous dynamics have demonstrated that PEX syndrome affects not only the conventional outflow pathway (PEX trabeculopathy) but also the uveoscleral outflow pathway, resulting in an elevated IOP (Gharagozloo et al., 1992; Johnson et al., 2008). The PMS creates an additional bleb‐based outflow but was designed to prevent NH, assuming a constant aqueous humour production (Ibarz Barberá et al., 2021; Pinchuk et al., 2017). Although aqueous flow is similar between unaffected eyes and eyes with PEX syndrome, the flow is slightly lower (15%) in eyes with PXG (Johnson et al., 2008; Johnson & Brubaker, 1982). These findings indicate that aqueous humour production is negatively affected by PEX cyclopathy, but to a lesser degree than outflow resistance, since IOP is elevated in PXG. In addition to a reduced baseline aqueous humour production in PXG, studies have demonstrated a reduced ocular blood flow in the presence of PEX (PEX vasculopathy) that could contribute to a higher transient postoperative impairment of humour production (Dayanir et al., 2009; Yüksel et al., 2001). These factors could explain why PXG resulted in higher rates of NH following PMS implantation in our study.

Another clinically relevant finding in our study is that PXG was an independent risk factor for CE following PMS implantation. This aligns with previous reports regarding trabeculectomy (Iwasaki et al., 2020) and AGV implantation (Shin et al., 2020). We found that the ORs for PXG as a predictor of NH and CE were similar (2.075 and 2.063). Thus, higher rates of CE might be an indirect consequence of higher rates of NH in PXG eyes. However, the degree of the mean postoperative IOP decrease was considered and controlled for in the final models, and PXG was found to be an independent risk factor without evidence of multicollinearity with the variable of IOP drop. Previous reports have suggested that PEX deposits in the extracellular matrix might cause a higher structural vulnerability of the choroid of PXG eyes, resulting in a susceptibility of the suprachoroidal space to deform in the presence of NH, which could explain our findings (Shin et al., 2020; Vazquez & Lee, 2014). In line with this, our analysis found that PXG was a risk factor for persistent CE requiring hypotony intervention. Greater impairment of the blood–aqueous barrier in PXG eyes could also be an additional explanation of our findings and the previous reports by Shin et al. (2020) and Iwasaki et al. (2020) (Johnson & Brubaker, 1982). In the subgroup of eyes with postoperative CE, 38% in the POAG group required interventions compared with 59% in the PXG group. Additionally, the CE duration was twice as long in the PXG eyes compared with the POAG eyes despite higher rates of interventions. These findings demonstrate that CE is not only more frequent but also more persistent in patients with PXG. However, the median number of AC injections in the patients with CE that required intervention as well as the frequency of intraluminal suture stenting to treat CE was comparable between the PXG and POAG subgroups, and all cases of CE resolved within 3 months of onset.

Our analyses showed older age as another independent risk factor for CE in the PXG and POAG eyes (adjusted OR = 1.046 per increasing year). This result agrees with previous reports on trabeculectomy (OR = 1.028) (Haga et al., 2013) and AGV implantation (OR = 1.05) (Shin et al., 2020). According to the authors, older age, which is associated with more fragile vascularity and connective tissue, could allow for more rapid accumulation of fluid in the suprachoroidal space. In our study, systemic variables such as antiplatelet or anticoagulant drugs, and especially arterial hypertension, did not influence the rate of hypotony, in contrast to the findings of Shin et al. (2020) who reported arterial hypertension as a risk factor.

We also found that a hyperopic refractive error was an independent risk factor for CE and CE requiring intervention in our cohort of PXG and POAG eyes. This finding aligns with previous reports that found hyperopia to be a risk factor for recurrent CE after trabeculectomy (Berke et al., 1987) and a shorter axial length as a risk factor for CE following AGV implantation (Fu et al., 2019). Fannin et al. (2003) reported that a myopic refractive error was protective against CE and a risk factor for hypotony maculopathy following trabeculectomy (retinal or choroidal folds within the arcades). Additionally, the presence of CE was associated with a decreased risk of hypotony maculopathy and vice versa, which was first clinically observed by Gass (1972) and speculated to be related to scleral thickness and rigidity as well as structural variations in the choroid and its vessels (Fannin et al., 2003; Saeedi et al., 2014). Axially myopic eyes have a thinner choroid and sclera, making them more likely to fold in the presence of NH, whereas axially shorter eyes with thicker sclera tend towards choroidal expansion (Saeedi et al., 2014). We did not find funduscopic signs of hypotony maculopathy in any patient, but subclinical asymptomatic hypotony maculopathy could be underdiagnosed because not all patients had an OCT scan during the early postoperative phase. We plan to investigate the presence of hypotony maculopathy in an OCT‐based study and report our findings in the future. Notably, a retrospective study found a longer axial length to be a risk factor of NH (IOP of <7 mmHg) within 3 months following XEN‐45 implantation (Galimi et al., 2023). However, the cohort was small, the study did not include multivariable risk factor analyses, and the provided data did not indicate differences in clinically significant hypotony with CE.

Male sex was another risk factor for the development of postoperative CE in our study (adjusted OR = 2.237). Similarly, male sex has been reported to be a risk factor for clinical hypotony following deep sclerectomy (Rabiolo et al., 2021) and CE following AGV implantation (OR = 2.77) (Ercalik et al., 2022). However, the exact mechanisms leading to a higher susceptibility for postoperative CE in male patients remain unclear. Male sex has also been reported as a risk factor for hypotony maculopathy (Fannin et al., 2003).

Experimental and theoretical flow testing demonstrated that the pressure drop across the PMS would not be large enough to avoid NH unless resistance to outflow is provided by surrounding tissue, which is increasingly provided by a developing filtering bleb in later maturation stages (Ibarz Barberá et al., 2021). In line, we found that NH prevalence was highest on day 1 and decreased by more than half until day 14. We found that CE development followed NH, and that CE prevalence increased until day 14, while NH decreased. The mean IOP reduction during the first two postoperative weeks was a risk factor in our multivariable analysis regarding the development of CE, agreeing with reports by Iwasaki et al. (2020) and Shin et al. (2020). A higher drop in IOP in this early postoperative period is reflective of lower postoperative IOP levels that increase vulnerability to develop CE, however not defined by a cut‐off value of <6 mmHg but on an individual level (Rabiolo et al., 2024). Our findings highlight the clinical importance of performing follow‐ups on days 2 and 14 post‐PMS implantation and performing a complete fundus examination, since NH is a non‐reliant surrogate of CE.

The number of preoperative IOP‐lowering agents was found to increase the risk of CE requiring intervention in our study. A higher number of agents might be associated with the application of topical aqueous suppressants. Ocular and systemic applications of aqueous suppressants have been reported as a risk factor for recurrent CE (Berke et al., 1987). However, we did not find the use of oral acetazolamide in the preoperative period (prior to the 2 weeks before surgery at the time of indication for surgery) as a risk factor for hypotony outcomes in the univariate analysis (Table S1).

Although we found no influence of CE or CE requiring intervention on the 6‐month visual outcomes, the eyes treated with CE interventions more frequently underwent bleb revision surgery, indicating a possibly detrimental effect on long‐term bleb function. This agrees with Benson et al. (2005), who found a decreased 5‐year survival rate of trabeculectomy blebs in the case of early, clinically significant hypotony. Other studies have not indicated differences in success rates between eyes with and without CE 6 months after AGV implantation (Fu et al., 2019) or 24 months after trabeculectomy or AGV implantation (Ercalik et al., 2022). However, these studies did not investigate the influence of hypotony interventions on the success rates of trabeculectomy and AGV implantation. In our study, the visual outcomes and bleb revision rates between the patients without CE and eyes with self‐limiting CE were comparable, and only those with CE requiring intervention underwent bleb revision surgery more frequently. Treating early postoperative hypotony with a viscoelastic injection into the AC reduces outflow into the bleb, thus increasing IOP to resolve CE. However, this disruption of early outflow into the bleb could interfere with bleb formation and might explain the higher rates of bleb revisions in our study.

Hypotony complications such as suprachoroidal bleeding in our study (1 PXG eye) were less common than those reported after trabeculectomy (up to 3%) or glaucoma drainage implant surgery (up to 2%) (Schrieber & Liu, 2015).

Our study has several limitations. First, it adopted a retrospective design, making it inherently biased. Second, while the number of patients and eyes included in this study was high, there were eyes with a follow‐up of <6 months. However, these were <9% of all eyes and were accounted for in the analyses. All other eyes were meticulously followed up by the same surgeon.

In conclusion, our study found that the PXG patients had a higher probability of developing CE following standalone unmodified PMS implantation and required hypotony intervention and subsequently bleb revision surgery more frequently than the POAG patients. Although all cases of CE resolved, its occurrence and intervention might have negative implications for long‐term bleb function. The diagnosis of PXG, higher age, hyperopia, the mean IOP drop within 2 weeks, and male sex were independent risk factors for clinically significant hypotony with CE. A diagnosis of PXG, hyperopia, and the number of preoperative IOP‐lowering agents were risk factors for CE requiring intervention.

Because the occurrence of clinically significant hypotony with CE increases the risk of possibly devastating sequelae and of bleb failure, we decided to perform primary intraluminal PMS stenting in all patients with PXG and high hyperopia in our clinical practice. This has been demonstrated to reduce the rates of hypotony in myopic eyes and eyes with mixed glaucoma diagnoses (Lupardi et al., 2023; Verma‐Fuehring et al., 2024). However, the long‐term implications of primary intraluminal PMS stenting on safety and success compared with standard PMS implantation have not yet been reported. We are convinced that our findings will help clinicians gauge the risk of hypotony following standard unmodified PMS implantation and guide patient selection and decision‐making regarding prophylactic intraluminal PMS stenting.

## CONFLICT OF INTEREST STATEMENT

EN has received a travel grant from Santen. BV has received speaker honoraria from Santen. IK, IS, DAM, and CJW declare no conflicts of interest.

## Supporting information


Figure S1.

Table S1.

Video S1.
