# Supplementary material for: Incidence, risk factors, and outcomes of numerical hypotony and choroidal effusion following PRESERFLO MicroShunt implantation
Source: Acta Ophthalmol. 2025 Oct 21;104(3):e239–50. doi: 10.1111/aos.70018 (PMC13058671; doi:10.1111/aos.70018)
Supplement: Supplementary file 1 — Figure S1. [file AOS-104-e239-s002.docx]

**Supplemental FIGURE 1** Sensitivity Analysis of Time-to-event analyses.

**

**

Kaplan–Meier estimates were used to analyze the cumulative incidence of numerical hypotony (IOP of <6 mmHg) (**A**), choroidal effusion (**B**) and choroidal effusion requiring intervention (**C**) and the duration of choroidal effusion (**D**) between the all eyes of patients with primary open-angle glaucoma (POAG; orange) and pseudoexfoliative glaucoma (PXG; blue). The 95% confidence intervals are represented in lighter orange and blue colors, respectively.
