# Supplementary material for: Incidence, risk factors, and outcomes of numerical hypotony and choroidal effusion following PRESERFLO MicroShunt implantation
Source: Acta Ophthalmol. 2025 Oct 21;104(3):e239–50. doi: 10.1111/aos.70018 (PMC13058671; doi:10.1111/aos.70018)
Supplement: Supplementary file 2 — Table S1. [file AOS-104-e239-s001.docx]

**TABLE S1.** Univariate GLLMs for numerical hypotony, choroidal effusion and choroidal effusion requiring intervention.

|  | **numerical hypotony** | | **choroidal effusion** | | **choroidal effusion requiring intervention** | |
| --- | --- | --- | --- | --- | --- | --- |
| **Variable** | ***p*-Value** | **OR, 95% CI** | ***p*-Value** | **OR, 95% CI** | ***p*-Value** | **OR, 95% CI** |
| Age, years | 0.813921 | 0.997, 0.976–1.019 | 0.2585449 | 1.065, 0.955–1.187 | 0.3963 | 1.074, 0.91–1.268 |
| Male sex | 0.962374 | 1.011, 0.652–1.566 | 0.0269232 | 1.964, 1.08–3.571 | 0.93067 | 0.885, 0.056–13.937 |
| Arterial hypertension | 0.862499 | 1.04, 0.67–1.614 | 0.6886622 | 1.491, 0.211–10.515 | 0.66935 | 1.901, 0.1–36.289 |
| Diabetes | 0.745943 | 0.905, 0.494–1.657 | 0.8705089 | 1.234, 0.099–15.43 | 0.88426 | 1.305, 0.036–47.089 |
| Antiplatelet prophylaxis | 0.846458 | 1.059, 0.592–1.897 | 0.0777586 | 2.027, 0.924–4.444 | 0.70892 | 1.841, 0.075–45.381 |
| Oral anticoagulants | 0.0587624 | 2.416, 0.968–6.03 | 0.4961312 | 2.542, 0.173–37.338 | 0.57108 | 2.76, 0.082–92.497 |
| Diagnosis of PXG (reference: POAG) | 0.0109295 | 1.959, 1.167–3.288 | 0.3254682 | 2.599, 0.387–17.442 | 0.32311 | 4.136, 0.247–69.111 |
| Refractive error, dpt spherical equivalent | 0.877324 | 0.996, 0.947–1.048 | 0.2247549 | 1.226, 0.882–1.703 | 0.20399 | 1.439, 0.821–2.522 |
| Pseudophakia | 0.947816 | 0.985, 0.627–1.547 | 0.5217113 | 1.997, 0.241–16.571 | 0.61612 | 2.304, 0.088–60.216 |
| Previous glaucoma surgery (reference: none) |  |  |  |  |  |  |
| Laser trabeculoplasty  Angle-based procedures  Trabeculectomy  Cyclo-/cryoablation  XEN-45 implantation | 0.228622  0.713851  0.643995  0.237083  0.815251 | 2.205, 0.609–7.993  1.152, 0.54–2.458  0.846, 0.416–1.721  1.708, 0.703–4.146  1.118, 0.44–2.841 | 0.2686842  0.4603567  0.7552123  0.6780334  0.9335889 | 6.239, 0.243–159.999  0.071, 0–78.878  0.603, 0.025–14.478  1.864, 0.099–35.246  1.177, 0.026–53.863 | 0.78323  0.7997  0.95528  0.79127  0.71716 | 3.78, 0–49216.95  0.127, 0–1042073.855  0, 0–1.86617572076E+195  0.167, 0–95882.581  6.575, 0–175108.67 |
| Number of preoperative medications | 0.350009 | 1.089, 0.911–1.302 | 0.3195825 | 1.671, 0.608–4.59 | <0.0001 | 2.734, 2.651–2.819 |
| Preoperative necessity for oral acetazolamide | 0.23063 | 0.685, 0.369–1.272 | 0.4588856 | 2.203, 0.273–17.799 | 0.37769 | 3.598, 0.209–61.865 |
| Preoperative medicated IOP, mmHg | 0.0483754 | 0.969, 0.939–1 | 0.008213 | 1.224, 1.054–1.422 | 0.071055 | 1.149, 0.988–1.335 |
| Mean postoperative IOP drop (preoperative IOP – mean IOP [days 1, 2, 7 and 14]), mmHg | Associated with outcome |  | 0.0045233 | 1.273, 1.078–1.504 | 0.053881 | 1.169, 0.997–1.371 |
| Postoperative AC haemorrhage | 0.969418 | 1.011, 0.582–1.755 | 0.8715491 | 1.188, 0.147–9.595 | 0.75968 | 1.621, 0.073–35.746 |

AC, anterior chamber; dpt, dioptre; POAG primary open-angle glaucoma; PXG, pseudoexfoliative glaucoma.
